# *Moringa oleifera* as a Natural Alternative for the Control of Gastrointestinal Parasites in Equines: A Review

**DOI:** 10.3390/plants12091921

**Published:** 2023-05-08

**Authors:** Mona Mohamed Mohamed Yasseen Elghandour, Aristide Maggiolino, Paulina Vázquez-Mendoza, Edwin Rafael Alvarado-Ramírez, José Cedillo-Monroy, Pasquale De Palo, Abdelfattah Zeidan Mohamed Salem

**Affiliations:** 1 Faculty of Veterinary Medicine and Zootechnics, Autonomous University of the State of Mexico, Toluca 50295, Estado de México, Mexico; 2 Department of Veterinary Medicine, University of Bari A. Moro, Valenzano, 70010 Bari, Italy; 3 Facultad Maya de Estudios Agropecuarios, Universidad Autónoma de Chiapas, Catazajá 29980, Chiapas, Mexico; 4 Multidisciplinary Academic Unit Mante, Autonomous University of Tamaulipas, El Mante 89840, Tamaulipas, Mexico; 5 Temascaltepec University Center, Autonomous University of the State of Mexico, Temascaltepec 51300, Estado de México, Mexico

**Keywords:** bioactive compounds, equines, gastrointestinal parasites, natural antiparasitics, *Moringa oleifera*

## Abstract

Studies have shown a wide variety of parasites that infect horses, causing major gastrointestinal damage that can lead to death, and although the main method of control has been synthetic anthelmintics, there are parasites that have developed resistance to these drugs. For generations, plants have been used throughout the world as a cure or treatment for countless diseases and their symptoms, as is the case of *Moringa oleifera*, a plant native to the western region. In all its organs, mainly in leaves, *M. oleifera* presents a diversity of bioactive compounds, including flavonoids, tannins, phenolic acids, saponins, and vitamins, which provide antioxidant power to the plant. The compounds with the greatest antiparasitic activity are tannins and saponins, and they affect both the larvae and the oocytes of various equine gastrointestinal parasites. Therefore, *M. oleifera* is a promising source for the natural control of gastrointestinal parasites in horses.

## 1. Introduction

Equines are used mainly as draft animals to till the land, as driving force attached to carriages, and as a means of transportation, especially in peri-urban and rural areas of various countries [1], for which they play an important role in the livelihood and economy of people worldwide. Despite this, the welfare of these animals is low, and their health is poor, and among the problems of greatest and most important concern is infection by gastrointestinal parasites [2,3]. According to various studies, nematodes are the most abundant group of gastrointestinal parasites within any equine population [4], and many of these nematodes have been associated with colic, frustration, and reduced anxiety and animal growth rates [5,6]. In addition, in the case of young and immunocompromised horses, they are also associated with diarrhea, abdominal pain, and can even cause death [7], reasons for which they are considered the main health threat to equine welfare [8]. For this reason, the constant monitoring and control of these parasites is an important aspect to minimize the negative impacts and guarantee a healthy and productive life in the animals [9,10].

In horses, parasite control is frequently carried out through anthelmintic treatments based on drugs of synthetic origin [11]. However, the timing and frequency of treatments, prolonged use of the same active ingredient, insufficient dosages, and unauthorized use of anthelmintic drugs are some of the factors that have led to decreased efficacy of synthetic anthelmintics and the development of different mechanisms in parasite populations to generate resistance to anthelmintics [12,13]. For this reason, this has become an important problem in veterinary medicine and, consequently, in recent years, research focused on finding new control alternatives has increased, and among the most promising is the use of plants, specifically those that present high concentrations of bioactive compounds [14]. In plants, these compounds are produced for various physiological functions, such as defense against pathogen attack and protection against abiotic stress, but within the organism of humans and animals, they produce medicinal effects [15]. Therefore, there are plants that have been used throughout the world and from generation to generation as a cure or treatment for countless diseases and their symptoms since it has been shown that they have antibacterial, anthelmintic, and insecticidal properties [16,17].

*Moringa oleifera*, commonly called “Moringa”, is a plant native to the western and sub-Himalayan region, India, Pakistan, Africa, and Arabia, currently cultivated in the Philippines, Cambodia, Central, North and South America, and the Caribbean islands [18], and is widely distributed in tropical and subtropical regions of the world, including Ghana, Uganda, Egypt, Sierra Leone, the Philippines, Nicaragua, Kenya, and Haiti [17]. Moringa belongs to the Moringaceae family, which is made up of 14 species worldwide, and of which Moringa is the most studied with respect to its phytochemical profile, especially the identification of bioactive compounds with therapeutic potential and their possible medicinal uses [19]. On the other hand, this plant is of important pharmaceutical value, thanks to the fact that its leaves, roots, seeds, fruits, and flowers have anti-inflammatory, antiparasitic [20], antidiabetic, antioxidant, immunomodulatory, anticancer, antihypertensive, and antimicrobial properties, which are of great importance for both humans and animals [21], and several analyses suggest that it has antiparasitic effects against *Plasmodium*, *Trypanosoma*, and *Leishmania* [20,21]. In addition, it is a species that can grow in adverse environments and with little rainfall since it can store enough water in its roots to tolerate prolonged periods of drought [22]. Therefore, the objective of this work was to document the main gastrointestinal parasites in horses, their effects on animal health, and the resistance they have presented to synthetic drugs, as well as to compile research involving the use of plants with antiparasitic activity and propose *Moringa oleifera* as a natural antiparasitic.

## 2. Main Equine Gastrointestinal Parasites

In horses, gastrointestinal parasitism is often associated with poverty in developing countries [23], but this is not the only factor influencing parasite incidence. In general, all equines are at risk of gastrointestinal parasitism [24], even from the first hours of life, due to a weak immune system [25,26], although animals that are under grazing conditions are more susceptible [27] since pastures can provide adequate conditions for the development of parasite larvae that are expelled by animals through feces [28], so these animals tend to have higher infection rates [26].

Although horses harbor many species of gastrointestinal parasites such as nematodes, protozoa, and multicellular cestodes, the diversity of the parasites varies depending on the age of the animal, with the youngest (<2 years) presenting the greatest diversity and parasite load in comparison with adult animals, which generally have already acquired some degree of immunity [25]. Table 1 shows the main most prevalent parasite species and the regions in which they have been reported.

**Figure 1:**
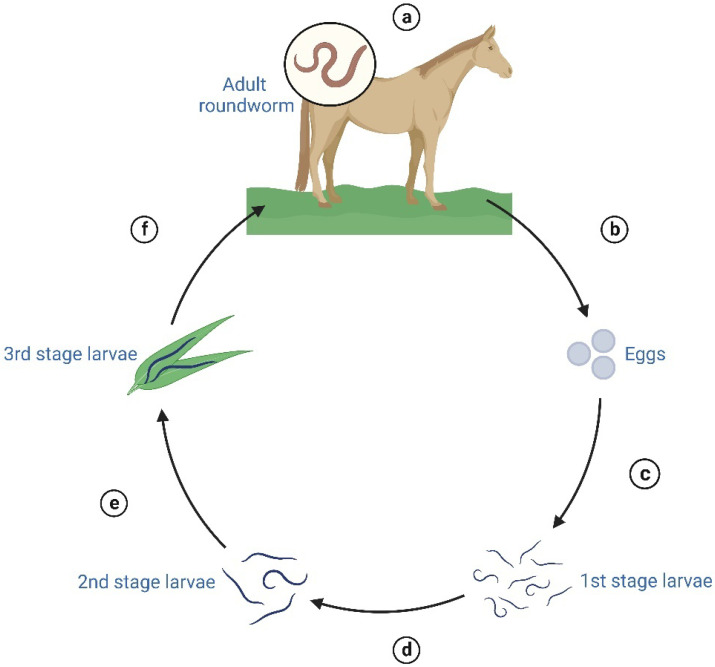
Life cycle of gastrointestinal nematodes: (**a**) adult females lay eggs in the host’s gastrointestinal tract, (**b**) eggs are shed by the host in feces, (**c**) first instar larvae remain in the bundles, (**d**) first instar larvae pass to second instars in feces, (**e**) second instar larvae become infective third instar larvae and migrate to vegetation, and (**f**) in the infective third instar larvae stage, they are ingested by the host and finally become adults within the cecum/colon.

As shown in Table 1, among the gastrointestinal parasites that infect horses, helminth parasites, especially nematodes, are the ones that have been reported most frequently and that present the greatest diversity of species, and it has been reported that they can constitute more than 75% of the total parasitic fauna in horses [44]. Equines can be infected by approximately 83 species of nematodes, including species of the *Strongylus* (large strongyles) and *Parascaris* genera and cyathostomines (small strongyles), the latter being considered the most important gastrointestinal parasite due to its prevalence, resistance to benzimidazoles and pyrantel, as well as its pathogenic effects [7,45]. Gastrointestinal nematodes are transmitted by pastures. The eggs hatch, and the larvae develop in the third infective stage in the environment, depending on exogenous factors such as atmospheric temperature, rainfall, and the type of terrain in which the animals are found (grass available, soil type, etc.; Figure 1) [45]. Because their cuticle acts as a protective layer, third instar larvae can tolerate freezing and desiccation for a period of time, while first and second instar larvae are less tolerant of these conditions [46]. Therefore, the infectivity of pastures with cyathostominae parasites depends to a large extent on environmental conditions [47].

In various parts of the world, numerous species of cyathostominae have been reported to infect equines, making them the most common helminths, with more than 50 described species, and causing disease in equines of all ages, but they are more frequently observed as larval ciatostomosis in young animals [45,48]. In addition to the above, these parasites have been considered successful in horses due to their life cycle, in which the early larvae of the third stage encyst within the intestinal mucosa, allowing them to survive toxicity of anthelmintics, except for moxidectin [45,49]. Unlike strongyles, parasites of the genus *Parascaris* spp. develop in ovo until the third larval instar, and during this time, they are protected from environmental conditions by the thick eggshell [50]. Under favorable conditions, such as moist soil, these eggs can survive for several years [51]. Infection by *Parascaris* spp. is produced by oral absorption of eggs that contain third stage larvae, a stage in which they are highly pathogenic in foals and yearlings and are sporadically found in adult horses [51,52].

On the other hand, researchers have reported that, as shown in Table 1, there is a high incidence in endemic areas of America, where the prevalence of equine cutaneous leishmaniasis infection exceeds that of humans, with a prevalence of 5, 3, and 28% of parasitic lesions in humans, dogs, and donkeys, respectively [53]. A serious disease that occurs in horses and other members of the equine family is equine piroplasmosis, which is caused by ticks that transmit the intraerythrocytic protozoa, *Theileria equi* and *Babesia caballi*, leading to anemia as a primary clinical result [39,54]. Infectious carriers are not always symptomatic, which means that there is a risk in non-zootic areas [54,55].

Table 1 shows that the European continent has a high prevalence of parasitic diseases in horses. An example is the countries of France and Italy, where since 1975, there have been several outbreaks of human trichinellosis as a consequence of the consumption of raw or undercooked horse meat [56,57]. The intestine constitutes the Trichinella’s initial point of contact with its host, and the duration of the enteral phase and the number of newly hatched larvae produced by females in the host’s intestine determine the duration of the disease, which stimulates a local inflammatory reaction culminating in death expulsion of intestinal worms [58]. On the other hand, *Oxyuris equi* is a common parasite of equines with worldwide distribution, which follows a direct life cycle with adults and inhabits mainly in the right dorsal colon [37], while *Sarcocystis neurona* is a pathogen that is housed in the central nervous system of horses and is considered the main cause of protozoan myeloencephalitis [41,59].

Cestodes occur in horses all over the world, so they are not considered endemic parasites of any specific region, and it has been reported that they use numerous species of oribatid mites that remain in pastures as intermediate hosts, so they are accidentally ingested by animals during grazing. In equines, it has been reported that they can only be infected by three species: *Anoplocephala perfoliata*, *Anoplocephala magna*, and *Anoplocephaloides mamillana* (syn. *Paranoplocephala mamillana*) [43], and of these, *A. perfoliata* is the most frequent, while the other two species have been observed sporadically [60]. Like most cestodes, both *A. magna* and *A. mamillana* house in the small intestine, whereas *A. perfoliata*, unlike these species, has a preference for the area around the ileocecal junction in the cecum [43].

## 3. Impact of Gastrointestinal Parasites on the Health of Equines

As has been observed in other herbivorous animals, equine gastrointestinal parasitism also affects the health and well-being of the animals, especially in young horses (less than 2 years of age) and is a risk factor for intestinal diseases [10]. One of these diseases is equine piroplasmosis, which is a consequence of the single or simultaneous infection of the blood apicomplex intracellular parasites *Theileria equi* and *Babesia caballi* [39], and some of the symptoms that have been shown in infected animals that presented severe acute disease are high fever, lethargy, anorexia, peripheral edema, splenomegaly, hemolysis, tachycardia, pigmenturia, and occasionally death [54,61]. In females, this parasitic disease decreases reproductive capacity and causes transplacental transmission, leading to abortion, fatal neonatal piroplasmosis at birth, or the offspring being born as subclinical carriers of the disease [62,63].

The equine bloodworm, *Strongylus vulgaris*, is considered the most pathogenic gastrointestinal parasite and is also commonly known as “the horse killer” [64]. Various investigations have shown that the clinical disease caused by this parasite is most frequently characterized by peritonitis [65] and that this condition was associated with positive anti-*S. vulgaris*, which confirmed that this parasite can cause the death of animals [66]. This is due to the migration of the larval stages carried out by this parasite within the cranial mesenteric artery, which causes inflammation and occlusion of the arteries, thus causing intestinal infarction and septic peritonitis [65]. Another disease is protozoal myeloencephalitis, which occurs when *Sarcocystis neurona* invades the central nervous system of the horse, causing asymmetric muscle atrophy and ataxia [41,59]. Gasterophilus are obligate parasites adapted to a larval life in the intestinal tract of horses and are the cause of gasterophilosis, a disease that causes lesions such as destruction of the gums, ulcerations, peritonitis, anemia, severe weakness, and blockage of the gastrointestinal tract, or even the death of the hosts [67]. Like other nematodes, small Strongyles have a remarkable pathogenic capacity upon entering the intestine and can cause clinical symptoms such as lethargy, sudden weight loss, wasting, and diarrhea [29]. In equines, *Parascaris equorum* represents a great threat, especially for young horses, with a prevalence ranging from 31 to 61% [32]. In addition, *P*. *equorum* infection is associated with lethargy, loss of appetite, decreased weight gain, hypoproteinemia, cough, and runny nose, especially in young horses [68], even causing death due to intestinal impaction or rupture [52].

Equine cyathostomins are parasites that can cause the critical disease called larval cyathostomy, a potentially fatal generalized typhocolitis [29], while Leishmania, an opportunistic parasite, is less severe in equines than in other hosts. It is not considered a deadly parasite, but the main symptoms reported are development of nodules in areas such as eyes, snout, neck, pinna, scrotum, and legs, and no visceral lesions have been described to date [69]. In the case of *Oxyuris equi*, the clinical signs are varied, but in severe cases, skin abrasions have been observed in the perineal region, and the eggs are quite resistant, so much so that they can persist in the perianal region and the environment for relatively long periods of time [59]. In younger horses, the small intestinal roundworm, *Parascaris equorum*, may represent a latent health risk, as the signs of the disease can be both respiratory and intestinal [68]. Although Oxyuroidea nematodes, or roundworms, do not represent a significant threat to equine health, female *O*. *equi* protrudes from the animals’ anus and deposits eggs in the perineal area, which becomes irritating to the host and, consequently, to horses. They rub the head of the tail and rump against fixed objects, causing local damage to the skin, fur, and tail [60]. In cestodes, unlike the other two species, *A. perfoliata*, in addition to being associated with various forms of colic, also causes pathological reactions around its attachment site, characterized by hyperemia, thickening of the mucosa, and necrotic ulcers, which explains why only this species is associated with clinical diseases in horses [43,70].

## 4. Resistance of Gastrointestinal Parasites to Synthetic Anthelmintics

For decades, the control of gastrointestinal parasites has been based on the application of synthetic anthelmintics, and for horses, there are three classes of broad-spectrum anthelmintics: benzimidazoles (fenbendazole and oxibendazole), tetrahydropyrimidines (pyrantel), and macrocyclic lactones (ivermectin and moxidectin) [27]. Although for years these drugs were a successful control method due to the effectiveness they demonstrated in reducing the parasite load [71], frequent use and inadequate quantity caused their efficacy to decrease [72], and the parasites developed resistance to anthelmintics [2,73].

The anthelmintic resistance of parasites has been defined by various authors as the decrease in the effectiveness of a drug to inhibit the growth and cause the death of the parasite when applied in therapeutically recommended doses [74]. Similarly, resistance is considered to be the loss of sensitivity of a parasite to a drug that was previously lethal to it [75]. According to Nipane et al. [76], there are three types of anthelmintic resistance: cross resistance, lateral resistance, and multiple resistance. Cross resistance is in which parasites are able to tolerate therapeutic doses of an anthelmintic that has a different mechanism of action, whereas lateral resistance is a condition in which resistance is due to selection for another anthelmintic that has a different mechanism of action. Similar action and multiple resistance occur when a parasite population exhibits cross and lateral resistance.

Four possible mechanisms by which equine parasites increase their resistance to anthelmintics have been identified: (i) activation of pre-existing alleles for resistance to toxic compounds, (ii) spontaneous mutations before or at the time of anthelmintic exposure, (iii) frequent mutations for the reappearance of alleles that provide resistance, and (iv) host migration of resistant alleles spread through new populations [12,26,77,78]. However, it is not ruled out that anthelmintic resistance may be the result of the selection of a parasite subpopulation that is capable of resisting the toxicity of drugs that were previously lethal to it and that this subpopulation selects specific genes that allow it to survive, which are believed to mutate and are responsible for the development of resistance [79]. Furthermore, once the parasite develops resistance within a parasite population, it persists for several years and passes the genes associated with resistance to its offspring [80,81]. Therefore, the control of gastrointestinal parasites in horses remains a complex challenge, and there is a need to seek new control alternatives [82,83].

## 5. Plant Bioactive Compounds for the Control of Gatrointestinal Parasites in Equines

The growing need to look for alternatives other than synthetic antiparasitics for the control of gastrointestinal parasites in herbivores, including equines, which, in addition to being effective, are also sustainable and do not cause toxicity in animals [84], has allowed plants to be considered as a viable option. This is because various plant species with antiparasitic activities have been identified [85,86], which even have the ability to partially or totally replace synthetic drugs and thus assist in the prevention of the antiparasitic resistance in parasites [87]. In addition, antiparasitic drugs made from plants are considered biodegradable and ecological [88], thanks to the fact that they are less likely to accumulate in the tissues of animals, and in some cases, they contribute to the mitigation of greenhouse gases, which reduces the impact of animals on the environment [89,90]. These benefits are mainly attributed to the presence of bioactive compounds in plants, such as alkaloids, flavonoids, tannins, saponins, coumarins, terpenes, lignans, and amino acids, which have been reported to have antimethanogenic and antiparasitic properties [86,91,92]. In the case of antiparasitic properties, it has been reported that the same plant species can present several mechanisms of action against parasites because they present a wide variety of bioactive compounds, which also decreases the probability that parasites develop antiparasitic resistance [93].

Despite the benefits that plants provide, there are few studies that have been carried out on their use in the control of gastrointestinal parasites in monogastric animals, particularly in horses, as highlighted by Scantlebury et al. [94]. In addition, the few studies carried out focus on parasites of the Strongylidae family, both in the cyathostominae and Strongylinae subfamilies, although the presence of cyathostominae is usually greater than strongylinae [95,96], as already mentioned above. In this sense, it has been reported that *Alectryon oleifolius* contains procyadinin A2, a compound that belongs to the group of condensed tannins and has demonstrated anthelmintic activity against cyathostomins in horses, since it is capable of completely inhibiting the development of these parasites from the egg to the third larval instar (L_3_) at a concentration of 50 µg mL^−1^, while at 25 µg mL^−1^, it inhibits larval migration [97]. In another study, they reported that of 37 plants evaluated, only *Acacia baileyana*, *Acacia melanoxylon*, *Acacia podalyriifolia*, *Alectryon oleifolius*, *Duboisia hopwoodii*, *Eucalyptus gomphocephala*, and *Santalum spicatum* were able to inhibit cyathostomin larval development by 100% compared to the control, with a concentration of 1400 µg mL^−1^, and they attributed it to the presence of tannins [98]. These authors also point out that *Duboisia hopwoodii* was the most potent plant in anthelmintic terms and that this was possibly due to the content of alkaloids, such as nornicotine and nicotine. Similarly, Peachey et al. [99] reported that when evaluating the potential use of nine plants for the control of cyathostomy eggs and L_3_ stage larvae, only *Acacia nilotica*, *Cucumis Prophetarum*, *Rumex abyssinicus*, *Allium sativum*, *Artemisia absinthium*, *Chenopodium album*, and *Zingiber officinale* showed anthelmintic activity. In the case of *Allium sativum*, its antiparasitic properties have been reported to be due to the presence of cysteine sulfoxide compounds (allicin, cycloalin, methiin, and isoaline) and *γ*-glutamylcysteine (*γ*-glutamyl-S-allylcysteine, *γ*-glutamyl -S-methylcysteine, and *γ*-glutamyl-S-t-propenylcysteine), and the synergy they form with each other for parasite control [100]. However, it has been shown that allicin, a molecule that belongs to the organosulfur group, is the most abundant (10 mg g^−1^ matter fresh) in *Allium sativum*, and that it is responsible for its characteristic odor when crushed, as well as the inhibition it exerts on the growth of *Babesia caballi* and *Theleria equi* at concentrations of 10 and 100 µM, respectively [100,101]. Contrary to this, Buono et al. [14] reported that in the short term (15 days), there is no effect of *Allium sativum* on the elimination of intestinal strongyle eggs in horses, while when evaluating the control of *Parascaris equorum* using five plants with anthelmintic properties, only *Artemisia dracunculus*, *Mentha pulegium*, and *Zataria multiflora* showed potential for larval control from the first instar to the fourth at concentrations greater than 100 µg mL^−1^, and *Eucalyptus camadulensis* and *Allium sativum* did not exert effective control over this parasite [102].

In an in vivo study, a 56.9% reduction of intestinal strongyle eggs was reported in *Equus asinus*, a species belonging to equines. This, with the use of an antiparasitic product derived from *Cardus mariano*, *Eucalyptus globulus*, *Gentiana lutea*, *Urtica urens*, and *Mallotus philippinensis*, and the anthelmintic activity, was attributed to the content of terpenoids, steroids, flavonoids, coumarins, and phenols that has been reported in these plants [103]. Instead, the saponins extracted from *Medicago arborea*, *Medicago sativa*, and the “Santiago” and “Angola” cultivars of the *Medicago polymorpha* species were able to inhibit the development and hatching of gastrointestinal parasite eggs by more than 80% in this same animal species [104]. Likewise, when evaluating *Albizia lophantha* against gastrointestinal nematodes in horses, 11 and 43% inhibition of egg hatching was achieved with concentrations of 80 and 120 µg mL^−1^, respectively, and was attributed to the presence of active compounds in the plant, especially tannins and total phenols [105]. On the other hand, Collas et al. [106] evaluated the effectiveness of *Achillea millefolium*, *Artemisia absinthium*, *Centaurium erythraea*, *Gentiana asclepiadea*, *Inula helenium*, and *Tanacetum vulgare* as ovicidal and larvicidal against nematodes in *Equus asinus*, and of all plants, Inula helenium showed the strongest effects at concentrations of 41 and 408 µg mL^−1^, respectively, and was attributed to the presence of sesquiterpene lactones and the inulin contained in this plant. Meanwhile, when evaluating the efficacy of *Onobrychis viciifolia* as an anthelmintic against larval development and strongyle egg hatching in horses, they reported that with 29% of this plant in the feces, it was possible to reduce the development of eggs of strongyles by 82% strongyles, while a concentration of 75 µg mL^−1^ decreased egg hatching by 37% [107]. This agrees with what was reported by Grimm et al. [108], who reported that *Onobrychis viciifolia* demonstrated an inhibitory effect on the motility of infective strongyle larvae, suggesting that it might have the ability to perturb strongyles at different stages of the life cycle. In the same way, it has been reported that the seeds of *Trachyspermum ammi* have anthelmintic activity against gastrointestinal nematodes in *Equus asinus*, thanks to the fact that the main component is thymol (54.6%), a compound that belongs to the group of terpenes and is considered as the active compound that causes the anthelmintic effect, although it also contains other compounds with anthelmintic properties and with which it acts in synergy [109].

## 6. Main Active Compounds of *Moringa oleifera*

*M. oleifera* is a plant whose parts are traditionally used for different purposes, but the leaves are generally the most used, particularly in human and animal nutrition, in traditional medicine and as a natural antiparasitic in ruminants [110,111,112] as they are rich in vitamins, carotenoids, polyphenols, phenolic acids, flavonoids, flavonoids, alkaloids, glucosinolates, isothiocyanates, tannins, and saponins [113]. Additionally, other parts of this plant contain an important mineral profile and are a good source of proteins, vitamins, *β*-carotene, amino acids, various phenolics, quercetin, βsitosterol, caffeoylquinic acid, and kaempferol [114].

Table 2 shows different bioactive compounds of *M. oleifera*, as well as their concentrations throughout the plant; however, it should be noted that differences in concentrations can be observed in the literature; this may be due to the variation between the age of the plants, exposure to the sun, the parts of the plant in which the concentrations were evaluated, geographical location, abiotic factors, nature of the solvents, or the method of extraction of the active compounds.

The compounds presented in Table 2 are all those that give *M. oleifera* its characteristic properties. Its leaves defend the plant from the effect of free radicals and pollutants due to its antioxidant activity as a result of the vitamins found in this part, especially the antioxidant effect of vitamin E [112,115]. Another of its main components is flavonoids, which are synthesized in the plant as a response to microbial infections, making them a good source of flavonoids, which are used as a potential compound against chronic diseases associated with oxidative stress [113]. Their main ones are Quercetin and Kaempferol, which have a higher antioxidant power than traditional vitamins [118]. Phenolic acids belong to the group of phenolic compounds derived from hydroxybenzoic acid and hydroxycinnamic acid, which are antioxidant, anti-inflammatory, antimutagenic, and anticarcinogenic compounds [117,121]. Several studies have described the potential use of moringa seeds, leaves, and gum extracts to control nematode parasites by containing potential secondary metabolites (tannins, saponins, alkaloids) responsible for more than 90% inhibition of egg hatching. Tannins have attracted the most attention for their antiparasitic effect on internal nematodes. Similarly, saponins are known for their excellent source of cytotoxic and anthelmintic compounds that justify their isolation and purification for the development of new drugs [128].

## 7. Antiparasitic Activity of *Moringa oleifera*

It is important to mention that there are currently no studies that have evaluated *Moringa oleifera* as an antiparasitic in horses, so this section describes the antiparasitic activity that *M. oleifera* could present in these animals. In addition, although it is known that the results of the studies may vary in response to the type of solvent used, what is intended is to demonstrate the antiparasitic activity of some bioactive compounds that contain *M. oleifera*, and therefore the type of extract is not mentioned. In this sense, the evaluation of medicinal plants has allowed the identification of some metabolites, such as alkaloids, phenols, and terpenes, that have been useful for the treatment of diseases caused by parasites in animals [129]. In the case of *M. oleifera*, it has been used to control malaria, trypanosomiasis, schistosomiasis, and filariasis [111], thanks to the bioactive compounds it presents [130]. In addition, it was reported that *M. oleifera* has powerful anthelmintic activity, these being the parasites that most attack equines (Table 1), added to the fact that it has ovicidal and larvicidal properties that hinder the hatching process of the eggs and larval development and converge in the death of the parasite [17].

In horses, it has been shown that bioactive compounds from plants can exert control over gastrointestinal parasites and that, in the case of nematodes (*Strongylus* sp., *Trichostrongylus axei*, *Strongyloides westeri*, and *Parascaris equorum*), the compounds with the highest activity against these parasites are phenols and tannins [105]. In this regard, it has been reported that the leaves of *M. oleifera* have the potential to combat nematodes [131], and according to what was reported by Mbogning-Tayo et al. [132], the leaves of mature trees can inhibit the development and hatching of the eggs in 92.8 and 99.0%, and cause the mortality of the first and second instar larvae in 98.8 and 100.0%, at a dose of 5 mg mL^−1^. Some researchers have attributed the antiparasitic activity mainly to the tannin content, but they are classified according to their chemical structure as follows: in condensed tannins, which are made up of complex compounds and are characterized by the presence of flavonoids, and in tannins, hydrolyzables that have a simple structure and contain smaller molecules, including gallic and ellagic acids [133]. Of both tannins, the hydrolyzed ones have greater antiparasitic activity than the condensed ones, especially gallic acid, which can inhibit almost 100% of gastrointestinal nematode eggs, at a dose of 1 mg mL^−1^ [134], and is capable of increasing its activity by entering into synergy with other compounds such as ellagic acid [135]. Although the mechanisms of action of tannins against gastrointestinal parasites are unknown with certainty, it has been suggested that, in the case of condensed tannins, it is possibly related to the number of hydroxyl groups since it is related to the capacity that the tannins present tannins to bind to proteins because this bond is formed by hydrogen bonds, a phenolic hydroxyl of the tannin (donor), and a carbonyl oxygen of the peptide bond in the protein (acceptor) [136]. In addition, tannins easily bind to proline, an abundant amino acid in the cuticle of nematodes, so when exposed to tannins, they suffer damage to the cuticle and intestinal surface [134,137]. However, tannin constituents can influence antiparasitic activity as some contain an acetal functional group, such as procyanidin A2, which increases the binding affinity for tannin proteins [97]. Similarly, it has been reported that they can interfere with energy generation by uncoupling oxidative phosphorylation, and it has been reported that they can act indirectly by improving the immune response of host animals [138]. However, it is possible that there is synergy between bioactive compounds, such as condensed tannins and two flavonoids, quercetin (flavonols) and luteolin (flavonoids), which, being in synergy, improve the inhibition of helminth sheathing [135].

In the family Cyathostominae, hatching of nematode eggs can be inhibited by phenolic acids (phenolic acids such as gallic acid, chlorogenic acid, caffeic acid, and rosmarinic acid) and flavonoids (rutin, luteolin, apigenin, and quercitin), which have cytotoxic effects, and the signaling pathways and gene expression appear, which leads to the death of larvae and adults of these parasites [139]. Likewise, these compounds can adhere to the egg membrane, which alters the permeability of the eggshell and interrupts osmosis or diffusion [133]. In addition, gallic acid has an affinity for proteins [140], and nematode eggs have a high content of glycoproteins on the outer surface of their cuticle, so the hydroxyl groups of gallic acid can be attached by links with eggshell proteins [133], and with them cause the inhibition of egg hatching. It has been reported that tannins also show high efficacy (100% inhibition) for the control of cyathostomin larval development in horses, the most abundant parasites in these animals, which is in agreement with other studies on the anthelmintic activity of tannins [97,98,141].

Saponins also play an important role in the antiparasitic activity of plants [142,143] since they have the ability to destabilize and penetrate the egg membrane, destroy content, and inhibit the activity of the enzyme responsible for hatching [144]. In addition, they can also induce anti-feeding behavior through paralysis of the alimentary system so that it weakens and detaches from the intestinal wall to finally be expelled through the feces [145]. However, the chemical structure of saponins is also key to antiparasitic efficacy since there are saponin constituents that present less antiparasitism than others [104]. Another bioactive compound in *M. oleifera* is terpenoids [146], which according to Imani-Baran et al. [109], in the seeds of *Trachyspermum ammi*, they were responsible for the control of nematodes in *Equus asinus*, which indicates that, in horses, the leaves of *M. oleifera* could cause a similar effect on nematodes.

Quercetin is a flavonol present in *M. oleifera*, which has been described as the most potent compound among polyphenols and with antinematocidal activity, since it can inhibit the hatching of eggs and the viability of nematode larvae [147]. Instead, caffeic acid is a bioactive polyphenol that is also present in *M. oleifera*, and is capable of inhibiting proteolysis, lipolysis, and suppressing the activation of matrix metalloproteinases, pathways, and enzymes that are activated by the nematode embryo before hatching [148]. It has been reported that the synergy of these two compounds is capable of inhibiting the hatching of nematode eggs by more than 90.0%, and it is attributed to the adhesion of these compounds and the damage they cause in structures of the cuticle of the eggs, which modifies the permeability of the membrane and allows the aggregation and infiltration of materials [147]. However, it has been reported that hatching is inhibited but not the embryonic process, so the mechanism of action is possibly the blocking of hatching in response to the increase in the thickness of the eggshell [147]. Despite the above, it is not ruled out that embryonic mortality inside the eggs is the cause of asphyxia and/or cellular toxicity associated with the accumulation of metabolic products [149].

**Figure 2:**
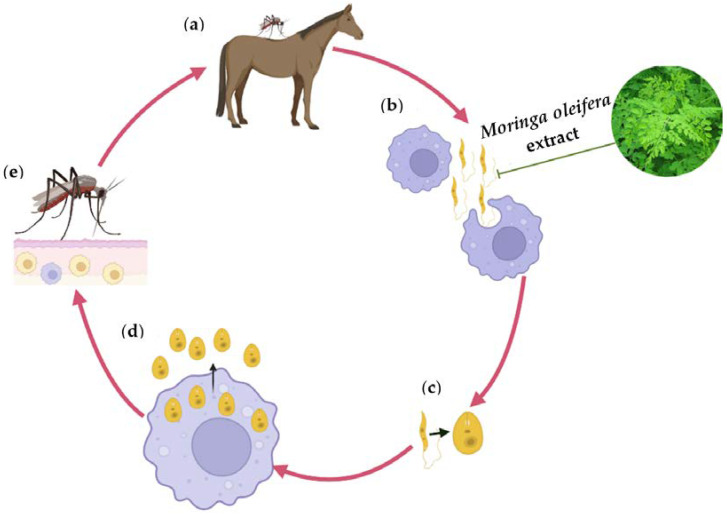
Life cycle of Leishmania in equines and *M*. *oleifera*-induced promastigote inhibition. (**a**) Insects inject promastigotes into the skin of equine, (**b**) Promastigotes are phagocytized by macrophages, (**c**) Promastigotes transform into amastigotes, (**d**) Amastigotes multiply and leave infected cells to infect new cells of various tissues, and (**e**) Sandfly takes blood with infected macrophages.

The antileishmanial activity of some plants has been attributed to the presence of compounds such as alkaloids, chalcones, triterpenoids, naphthoquinones, quinones, terpenes, steroids, lignans, saponins, and flavonoids [130]. In this sense, *M. oleifera* has shown that it has a high activity against *Leishmani donovani* and significantly reduces infected macrophage cells (86%), thus attacking both stages (promastigotas and amastigotas) of Leishmania [121,150]. Similarly, Allahverdiyev et al. [151] analyzed the antileishmanial effects of *M. oleifera* nanoparticles against parasites and observed that the growth and metabolic activity of Leishmania is inhibited as a result of the effects that *M. oleifera* causes on mitochondrial function through oxidative stress and the infectivity of promastigotes by impeding the survival of amastigotes within the host cells. It has also been reported that *M. oleifera* affects Leishmania through a change in the morphology of the promastigote, uncovering the loss of its flagellum and inducing a change in appearance from oval to circular [152]. These changes inhibit their mobility, preventing them from reaching immune system cells such as macrophages, phagocytes, and granulocytes, in which, as shown in Figure 2, endocytosis is necessary to have the necessary means to mature into amastigotes, a stage in which parasites can reproduce and continue to infect more cells in different tissues [152]. Furthermore, it has been reported that *M. oleifera* does not show cytotoxicity on macrophage cells, as it has been shown that there is no difference between treated and untreated, uninfected macrophage cells [153]. Despite the research that has been carried out on the different active compounds of *M. oleifera*, it is considered that the greatest antiparasitic effects are not provided by a single compound but by several since they act synergistically, as already mentioned.

## 8. Future Perspectives

Considering the usefulness of equines in the daily activities of human beings, the resistance that gastrointestinal parasites are generating to synthetic drugs in these animals, and the damage that these parasites cause to equine health, it is necessary to seek new alternatives of control. Undoubtedly, in this situation, bioactive compounds from plants offer a natural and environmentally friendly alternative, and in this current era, it is necessary to think about the future and the generation of ecological products. In this sense, *Moringa oleifera* is a plant that has demonstrated anthelmintic activity in ruminants, it has even been considered a candidate for the generation of new drugs, but in horses, it has not been evaluated as an antiparasitic, so scientific bases need to be generated that allow its use as a natural antiparasitic. This involves exploring various extraction methods (aqueous, methanolic, and ethanolic) to obtain bioactive compounds (tannins, flavonoids, phenolics, saponins, and alkaloids) with antiparasitic activity since there are compounds that have more affinity for some solvents than others and the antiparasitic potential depends on the extracted compounds [154]. In the same way, when defining the doses, it is necessary to consider the toxicity that each bioactive compound could cause in the animals and thus avoid adverse effects on their health, as well as evaluate in different types of parasites and larval stages and establish the mechanism of action that exerts on the control of parasites. In addition, it has been hypothesized that bioactive compounds from some plants do not exert direct anthelmintic activity on parasites but instead enhance the host animal’s immune system, thereby indirectly reducing the parasite load [103]. Therefore, the hematological profile of the animals should be included in the in vivo evaluations.

## 9. Conclusions

Given the need to generate an alternative for the control of gastrointestinal parasites that are presenting resistance to synthetic drugs in horses, *Moringa oleifera* represents an alternative that is not only natural, but also ecological for the control of these parasites. The main benefit of this plant is found in its leaves, which is the bioactive compounds that it presents, both in the region where it is native and in the places where it is cultivated. These compounds have demonstrated antiparasitic activity, and the main ones are flavonoids, phenolic acids, tannins, vitamins, and saponins. In addition, this plant is capable of adapting to a wide variety of edaphoclimatic conditions, even adverse conditions, which gives it an advantage over those plants that have antiparasitic activity but do not adapt to any region. Therefore, *M. oleifera* can be considered as a source of bioactive compounds for the generation of new drugs of natural origin for the control of gastrointestinal parasites in horses, and it can be cultivated in different regions for industrialization purposes. However, research is required to validate and understand its mechanism of action, with the aim of a safer and more correct application in animals.

## Figures and Tables

**Table 1:** Main species of parasites infecting equines, compared to the regions where they are most prevalent.

Type of Parasite	Predominant Parasite Species	Country with Highest Prevalence	References
*Nematode*	*Cyathostomum* *catinatum* *Cylicostephanus longibursatus*	France, Ukraine, United States, and Australia	[29]
	*C. nassatus*	Brazil	[29]
	*Strongyles* spp.	United States, Scandinavia, Sweden, Holland, Denmark, Ethiopia, and Kenya.	[30,31]
	*Caenorhabditis* *elegans*	Europe, North America, New Zealand, Australia, Ethiopia, and Saudi Arabia.	[32]
	*Trichinella*	Romania, France, and Italy	[33]
	*Gasterophilus* sp.	Belgium, Ireland, Poland, France, Germany, Morocco, Turkey, Saudi Arabia, Brazil, and China.	[34,35,36]
	*Oxyuris equi*	United States, New Zealand	[37]
*Protozoa*	*Leishmania*	Argentina, Brazil, Venezuela	[38]
	*Theileria equi*	Asia, Europe, Africa, and South America	[39]
	*Babesia caballi*	East and Southern Africa, Asia (especially China and Korea), Europe (Portugal, Spain, France, Italy, Hungary, and Romania), Chile, and Argentina.	[40]
	*Parascaris* sp.	Europe, North America, New Zealand, Australia, Ethiopia, and Saudi Arabia.	[32]
	*Sarcocystis neurona*	Argentina, Brazil, and North America.	[41,42]
*Cestode*	*Anoplocephala* *perfoliata* *Anoplocephala magna* *Anoplocephaloidess* *mamillana*	Spain, Poland, and Sweden	[43]

**Table 2:** Bioactive compounds of *Moringa oleifera*.

Type of Compound	Active Compounds	Part of Plant	Concentration	References
Vitamins	γ-tocopherol	Adult leaves	5.7 µg g^−1^	[115]
		Young leaves	27.8 µg g^−1^	
	Vitamin C	Fresh leaves	200 mg 100 g^−1^	[112]
		Dried leaves	18.7–140 mg 100 g^−1^	
	Vitamin A	Fresh leaves	11,300 (U.K.)	[116]
Flavonoids	Luteolin	Leaves	6.2 μg g^−1^	[117]
	Quercetin	Leaves	0.207 mg g^−1^	[118]
	Myricetin	Leaves	5.8 mg g^−1^	
	Kaempferol	Leaves	7.57 mg g^−1^	
	Epicatechin	Freeze-dried leaves	5.68 mg g^−1^	[119]
Phenolic acids	Caffeic acid	Freeze-dried leaves	0.536 mg g^−1^	[119]
	O-coumaric acid	Freeze-dried leaves	6457 mg g^−1^	
	Gallic Acid	Leaves	1034 mg g^−1^	[120,121]
	Chlorogenic acid	Leaves	0.018–0.489 mg g^−1^	
	Ellagic acid	Leaves	0.009–0.189 mg g^−1^	
	Phenols	Leaves	10 mg mL^−1^	[122]
	Ferulic acid	Leaves	128.2 µg g^−1^	[123]
	Rutin	Leaves	190 µg g^−1^	
Glucosinolates	4-(α-L- ramnopyranosyloxy)- benzyl	Freeze-dried young leaves	59.4 mg g^−1^	[124]
Tannins	Tannins	Dried leaves	12–20.6 g kg^−1^	[125,126]
Saponins	Saponins	Dried leaves	64–81 g kg^−1^	[127]

## Data Availability

Not applicable.
